# Supplementary figures and images for: Highly efficient scarless knock-in of reporter genes into human and mouse pluripotent stem cells via transient antibiotic selection
Source: PLoS One. 2018 Nov 29;13(11):e0201683. doi: 10.1371/journal.pone.0201683 (PMC6264506; doi:10.1371/journal.pone.0201683)

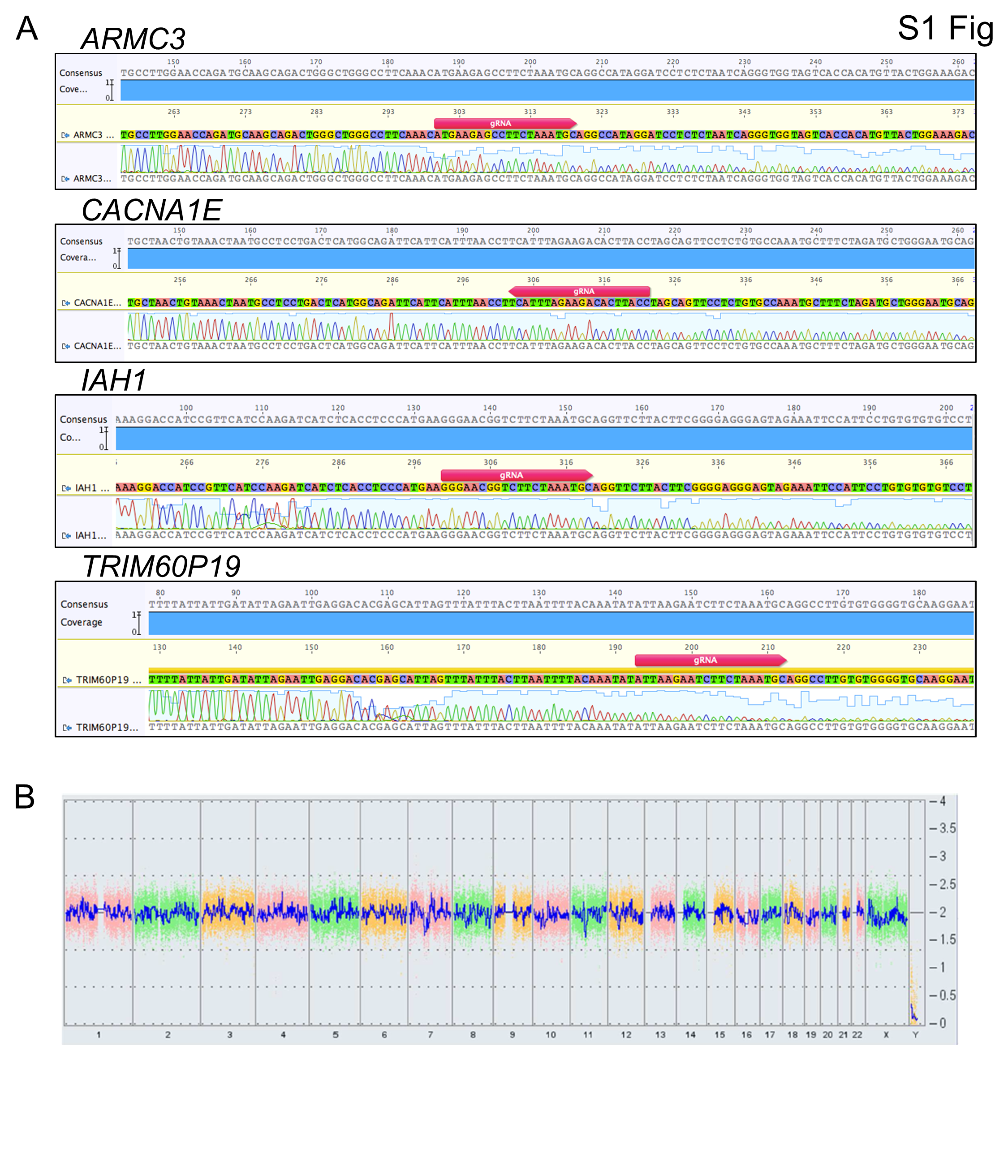

Supplement: S1 Fig — (A) The four most likely in-silico predicted off-target mutations for the BRN3B gRNA were sequenced and confirmed to be WT. (B) Karyotyping analysis using KaryoStat found no chromosomal aberrations. (TIF) [file pone.0201683.s001.TIF]
